# Human IgG_1_ Responses to Surface Localised *Schistosoma mansoni* Ly6 Family Members Drop following Praziquantel Treatment

**DOI:** 10.1371/journal.pntd.0003920

**Published:** 2015-07-06

**Authors:** Iain W. Chalmers, Colin M. Fitzsimmons, Martha Brown, Christine Pierrot, Frances M. Jones, Jakub M. Wawrzyniak, Narcis Fernandez-Fuentes, Edridah M. Tukahebwa, David W. Dunne, Jamal Khalife, Karl F. Hoffmann

**Affiliations:** 1 Institute of Biological, Environmental & Rural Sciences (IBERS), Aberystwyth University, Aberystwyth, United Kingdom; 2 Department of Pathology, University of Cambridge, Cambridge, United Kingdom; 3 Centre d'Infection et d'Immunité de Lille, Inserm U1019-UMR CNRS 8204, Institut Pasteur de Lille, Lille, France; 4 Vector Control Division, Ugandan Ministry of Health, Kampala, Uganda; George Washington University, UNITED STATES

## Abstract

**Background:**

The heptalaminate-covered, syncytial tegument is an important anatomical adaptation that enables schistosome parasites to maintain long-term, intravascular residence in definitive hosts. Investigation of the proteins present in this surface layer and the immune responses elicited by them during infection is crucial to our understanding of host/parasite interactions. Recent studies have revealed a number of novel tegumental surface proteins including three (SmCD59a, SmCD59b and Sm29) containing uPAR/Ly6 domains (renamed SmLy6A SmLy6B and SmLy6D in this study). While vaccination with SmLy6A (SmCD59a) and SmLy6D (Sm29) induces protective immunity in experimental models, human immunoglobulin responses to representative SmLy6 family members have yet to be thoroughly explored.

**Methodology/Principal Findings:**

Using a PSI-BLAST-based search, we present a comprehensive reanalysis of the *Schistosoma mansoni* Ly6 family (SmLy6A-K). Our examination extends the number of members to eleven (including three novel proteins) and provides strong evidence that the previously identified vaccine candidate Sm29 (renamed SmLy6D) is a unique double uPAR/Ly6 domain-containing representative. Presence of canonical cysteine residues, signal peptides and GPI-anchor sites strongly suggest that all SmLy6 proteins are cell surface-bound. To provide evidence that SmLy6 members are immunogenic in human populations, we report IgG_1_ (as well as IgG_4_ and IgE) responses against two surface-bound representatives (SmLy6A and SmLy6B) within a cohort of *S*. *mansoni*-infected Ugandan males before and after praziquantel treatment. While pre-treatment IgG_1_ prevalence for SmLy6A and SmLy6B differs amongst the studied population (7.4% and 25.3% of the cohort, respectively), these values are both higher than IgG_1_ prevalence (2.7%) for a sub-surface tegumental antigen, SmTAL1. Further, post-treatment IgG_1_ levels against surface-associated SmLy6A and SmLy6B significantly drop (*p* = 0.020 and *p* < 0.001, respectively) when compared to rising IgG_1_ levels against sub-surface SmTAL1.

**Conclusions/Significance:**

Collectively, these results expand the number of SmLy6 proteins found within *S*. *mansoni* and specifically demonstrate that surface-associated SmLy6A and SmLy6B elicit immunological responses during infection in endemic communities.

## Author Summary

Adult schistosome parasites can live in the human bloodstream for years without being adversely affected by the host immune response. Identifying which proteins are on the surface of the parasite and understanding how they contribute to long-term host/parasite relationships is an essential step in developing novel intervention strategies. Here, utilising a comprehensive bioinformatics approach to identify *Schistosoma mansoni* gene products sharing distinct surface-associated features including signal peptides, hydrophobic C-termini, disulfide bonds and uPAR/Ly6 domains, we identified eleven proteins of interest. These proteins, reassuringly, include three representatives previously found associated with the schistosome surface (here termed SmLy6A, SmLy6B and SmLy6D) as well as three novel members (SmLy6G, SmLy6H and SmLy6J). To identify if surface-associated SmLy6 members are recognized by *S*. *mansoni* infected individuals, we specifically examined antibody responses to SmLy6A and SmLy6B in an endemic human population. Our work expands the number of putative cell surface associated schistosome proteins and provides a greater understanding of the dynamics of antibody responses in endemic communities against two representatives.

## Introduction

Human schistosomiasis is estimated to affect more than 200 million people living in developing countries, with 120 million people symptomatic and 20 million suffering severe illness [1]. With a further 600 million individuals at risk of infection from *Schistosoma mansoni*, *Schistosoma haematobium* and *Schistosoma japonicum* (the three main human-infective species) and up to 70 million disability-adjusted life years (DALYs) lost annually, this neglected tropical disease (NTD) is one of the most important on the planet [2]. Schistosomiasis control is predominantly facilitated by mass drug administration (MDA) of praziquantel, a safe and potent chemotherapy developed in the late 1960’s [3]. However, mono-chemotherapy control of schistosomiasis raises the spectre of drug resistance [4] and highlights the need for developing an immunoprophylactic anti-schistosomal vaccine. Throughout the past several decades, progress in schistosome vaccine development has proved disappointing, with six promising candidates failing to induce sufficient protection in independent experimental models [5]. However, with the increased use of genomic, transcriptomic and proteomic methodologies to characterize schistosome biology, the number of novel and, perhaps more potent, vaccine candidates has vastly expanded [6–12].

Recent DNA microarray analyses [10] and sub-proteomic studies [11] have contributed to these efforts and led to the prioritization of several *S*. *mansoni* immunoprophylactic candidates. Amongst these, three (smp_081900, smp_019350 and smp_105220 –GeneDB.org identifiers) contained weak homology to Lymphocyte Antigen 6 (Ly6) proteins. Ly6 family members, such as the CD59 protein and urokinase/plasminogen activator receptor (uPAR) in humans, are extracellular, cysteine-rich proteins commonly tethered to the plasma membrane via a glycosylphosphatidylinositol (GPI) moiety [13,14]. All Ly6 family members contain one or more uPAR/Ly6 domains (Pfam identification code: PF00021; [15]), a 60–110 amino acid region that forms a characteristic “three-fingered protein domain” (TFPD) fold and contains eight or ten conserved cysteines [16]. This TFPD fold places Ly6 proteins as part of the larger TFPD protein superfamily, which also includes the transforming growth factor beta receptor (TGFβR) family and the snake α-neurotoxins family [16]. While each of these three protein families share the same core fold, they are differentiated by protein features (e.g. presence of C-terminal intracellular kinase domain in TGFβR family; [17]) and cysteine spacing (e.g. absolute conservation of C^1^-X-X-C^2^ spacing in the Ly6 family; [18]). Unlike the restricted phylogenetic representation of the snake α-neurotoxins, Ly6 genes have been found across both vertebrate and invertebrate phyla (*Homo sapiens* CD59; [19], *Drosophila melanogaster* rtv; [20] and *Caenorhadbditis elegans* odr-2 [21]). Functionally, Ly6 proteins have been involved in diverse roles ranging from septate junction formation in *D*. *melanogaster* [18], odorant response in *C*. *elegans* [21] and limb regeneration in salamanders [22]. In humans, the majority of the 27 Ly6 proteins [23] have unknown functions except for a few notable exceptions including the CD59 protein and uPAR [16]. CD59 is a cell surface glycoprotein found on most cells and functions to prevent complement-mediated lysis by binding C8α and C9 to prevent the formation of the complement membrane attack complex [24]. The triple uPAR/Ly6 domain-containing uPAR protein is a cell surface receptor for urokinase plasminogen activator (uPA), binding uPA to regulate its activity in extracellular matrix remodeling [25]. In schistosomes, proteomic analyses of the parasite tegument have identified two uPAR/Ly6 domain-containing proteins on the surface of the adult (initially named SmCD59a and SmCD59b; [11]) and another member identified within the tegumental fraction (smp_081900; [26]). However, subsequent analysis by Farias *et al*. has identified that these proteins are not functional orthologs of the human CD59 protein and are, rather, part of a larger set of *S*. *mansoni* uPAR/Ly6 domain-containing proteins containing seven members with unknown functions [27].

Here, we comprehensively re-analyse the *S*. *mansoni* genome to identify an expanded repertoire of eleven SmLy6 family members, SmLy6A-K, and describe the serological immune responses elicited by the two *S*. *mansoni* Ly6 family members initially named SmCD59a and SmCD59b (renamed in this study SmLy6A and SmLy6B) during infection in endemic communities. Specifically, we compare IgG_1_, IgG_4_ and IgE responses against SmLy6A and SmLy6B to SmTAL1 (a characterized non-surface tegumental protein) in a cohort of *S*. *mansoni* infected Ugandan males. Here, we detect evidence of anti-SmLy6 IgG_1_ (>IgG_4_>IgE) specific immune responses with IgG_1_ titers reflecting the differential abundance of SmLy6A and SmLy6B found throughout the schistosome lifecycle. Finally, we show that IgG_1_-specific SmLy6A and SmLy6B titers drop after praziquantel treatment. Together, our data collectively expand the number of putative cell-surface Ly6 family members found in *S*. *mansoni* and demonstrate that infected humans mount an immunoglobulin response against two representative members, which is strongly supportive of their surface localisation. These findings may contribute to future studies investigating the immunoprophylactic potential of the SmLy6 family.

## Materials and Methods

Unless otherwise stated, all chemicals and reagents were purchased from Sigma-Aldrich, United Kingdom.

### Ethics statements

All procedures performed on mice and rats adhered to the European Union Animals Directive 2010/63/EU and were approved by both Aberystwyth University’s animal welfare and ethical review body (AWERB, project license PPL 40/3700) and the French local ethics committee (CEEA Nord Pas de Calais). Ethical clearance for the Musoli cohort was obtained from the Uganda National Council of Science and Technology (ethics committee for Vector Control Division, Ugandan Ministry of Health) who approved the age of consent as 15 yr at the time of sample collection (2004/2005). Consent forms were translated into the local language and informed written consent was obtained from all adults and from the parents/legal guardians of all children. Parental consent was not sought for individuals between 15–18 yr old.

### Parasite material

The life cycle of a Puerto Rican strain (NMRI) of *S*. *mansoni* was maintained via routine passage through *Biomphalaria glabrata* snails and C57BL/6 mice (Harlan, United Kingdom) [28]. Mixed-sex schistosomula (cultured for 24-hr), miracidia and adult worms (7-week old) were obtained as previously described [29].

### Bioinformatic identification and characterisation of SmLy6 sequences

PSI-BLAST [30] searches were performed with five separate representative Ly6 proteins obtained from NCBI—*Homo sapiens* CD59 ([AAA60957]; [24]), *H*. *sapiens* uPAR ([Q03405.1]; [25]), *C*. *elegans* ODR-2 ([NP_001024089]; [21]), *D*. *melanogaster* Boudin ([NP_572373]; [18]) and *D*. *melanogaster* Rtv ([NP_572693]; [20]) against the *S*. *mansoni* RefSeq database (genome version 5.0) [31]. Selection of potential *S*. *mansoni* Ly6 homologues during PSI-BLAST searches required the protein sequence to contain three characteristic features of uPAR/Ly6 domains used previously to identify Ly6 proteins [18]: 1) presence of ten or more cysteine residues within a 60–110 aas region (lower and upper amino acid length defined using existing uPAR/Ly6 domains (Pfam domain: PF00021; [15])), 2) presence of an N-terminal motif C^1^-X-X-C^2^ (where C^1^ represents the first cysteine residue, X represents any amino acid and C^2^ represents the second cysteine residue) and 3) presence of a C-terminal C^10^-N motif (where C^10^ represents the tenth cysteine residue and N represents asparagine). Newly identified Ly6 homologues were incorporated into the search matrix until no more members could be identified, typically after five to six rounds of iterative searching. Then, these sequences were used as novel queries to identify any further homologues remaining in the *S*. *mansoni* genome. To identify putative orthologues for SmLy6A-K in the *S*. *haematobium* and *S*. *japonicum* genomes, tBLASTn searches using each of the SmLy6 family members was performed against the latest publically available gene predictions (searches performed 17-08-2013) from those genomes (downloaded from SchistoDB.net and GeneDB.org respectively).

Alignment of the SmLy6 family members and HsCD59 protein sequences was performed using MUSCLE software [32] with additional manual editing based on canonical residues implemented as necessary. The prediction of signal peptides was performed using the software SignalP 3.0 [33] and presence/absence of signal peptides was defined by the default Neural Network Dscore threshold of 0.43. Predicted hydrophobic regions in SmLy6 proteins were assessed using TMpred software [34] and potential GPI anchor sites were determined using the BIGPI server [35] with the site possessing the lowest e-value indicated.

### Cloning of SmLy6 sequences

*S*. *mansoni* total RNA was isolated [10] and used as templates to synthesise cDNA by reverse transcription as previously described [36]. SmLy6 sequences were confirmed by PCR amplification of cDNA obtained from either 7-week adult mixed-sex worms (SmLy6A-I) or miracidia (SmLy6J, SmLy6K) using Phusion proof-reading polymerase (Finnzymes). Nucleotide sequences for each SmLy6 family member are deposited in Genbank (accession numbers provided in Table 1) and primers used to amplify SmLy6 sequences by PCR are listed in S1 Table.

**Table 1:** SmLy6 family nomenclature and characteristics.

Family Name	Alternate Name(s)^a^	Gene Name^b^	Accession number^c^	Genome position^d^	Protein size (AA)^e^	Sh and Sj Ortholog^f^
SmLy6A	SmCD59a [11] SmCD59.1 [27]	Smp_019350	XM_002573337	Chr_1.SC_0010: 1429098–1435466	126	Sha_106850 (84%) Sjp_0098860 (50%)
SmLy6B	SmCD59b [11] SmCD59.2 [27] Dif 5 [50]	Smp_105220	XM_002570515	Chr_2: 23740934–23757067	124	Sha_200489 (93%) Sjp_0059750 (72%)
SmLy6C	SmCD59.3 [27]	Smp_081900.2	XM_002579231	Chr_1: 59144966–59146648	120	Sha_200766 (69%) No Sj homolog
SmLy6D	Sm29 [44]	Smp_072190	XM_002578361	Chr_1: 51486745–51491937	191	Sha_102899 (64%) Sjp_0041250 (47%)
SmLy6E	SmCD59.7 [27]	Smp_125250	XM_002572005	Chr_1: 38670211–38674024	129	Sha_106826 (76%) No Sj homolog
SmLy6F	SmCD59.4 [27]	Smp_166340	XM_002579233	Chr_1: 59132456–59139459	123	No Sh homolog Sjp_0078290 (53%)
SmLy6G	-	Smp_041550	XM_002575463	Chr_7.SC_0100: 1175980–1187625	146	Sha_108221 (70%) No Sj homolog
SmLy6H	-	Smp_074590	XM_002578587	SC_0144: 761831–769109	147	Sha_200688 (95%) Sjp_0078660 (75%)
SmLy6I	SmCD59.5 [27]	Smp_081920	XM_002579236	Chr_1: 59105712–59109795	124	Sha_300533 (73%) No Sj homolog
SmLy6J	-	Smp_158960	KP835575	SC_0167: 214022–218675	152	Sha_102600 (93%) Sjp_0023000 (85%)
SmLy6K	SmCD59.6 [27]	Smp_166350	XM_002579234	Chr_1: 59123155–59125102	114	Sha_109174 (80%) No Sj homolog

a) Nomenclature used in previously published literature

b) *S*. *mansoni* GeneDB ID

c) Genbank accession number for nucleotide sequence

d) position of gene in *S*. *mansoni* genome assembly version 5.1

e) Full length protein length before processing

f) percentage identity between proteins shown as % (only >45% shown).

### DNA microarray transcription profiles

Data from the 37,632 element *S*. *mansoni* long-oligonucleotide DNA microarray studies of Fitzpatrick *et al*. were interrogated to find the transcription pattern of SmLy6 family members in 14 life-cycle stages [10]. A full set of raw and normalized data is available via Array express under the experimental accession number E-MEXP-2094.

### Tertiary structure modeling

Tertiary structural modeling of SmLy6 family members was performed using the predicted mature protein sequences of SmLy6A-K produced by translating the sequenced transcripts and removing the amino acids encoding signal peptides and regions C-terminal to the GPI anchor.

Given the lack of significant sequence identity to any known structure and the small size, the structure of the protein was predicted using Rosetta [37] following the protocol described elsewhere [38] with the modification that only 1000 models were taken forward for full-atom minimization. Structural models were ranked using Rosetta’s energy function and the top 20 models were visually inspected to assess the convergence of the models towards a similar fold. Models were visualized using PyMOL (DeLano Scientific LLC).

### Expression and purification of recombinant proteins in *Escherichia coli*

Oligonucleotide primers incorporating *Xba*I and *Xho*I restriction sites were used to amplify SmLy6A (forward primer: 5'–TCTAGAATGCATCGTTGTTATGTG—3'; reverse primer 5'–CTCGAGTGTTTGTGTACCAGAA—3') and SmLy6B (forward primer: 5'—TCTAGAATGATAAAAAATAAGAAAGTC—3'; reverse primer 5'—CTCGAGATGTTTAGGTGATGC—3') using Platinum Taq DNA high fidelity polymerase (Invitrogen) and a pJET1.2/blunt plasmid (Fermentas) containing either SmLy6A or SmLy6B full-length open reading frames as templates. The primers were designed to amplify the SmLy6 sequences encoding the predicted mature protein of SmLy6A (His28—Thr104) and SmLy6B (Ile20—His101). These products were inserted into the *Xho*I and *Xba*I sites of a modified pET30a expression vector (Novagen) designed to add a C-terminal 6xHis tag. Both pET30a/SmLy6A and pET30a/SmLy6B were sequenced (IBERS sequencing facility, Aberystwyth University) before use to confirm correct reading frame and encoded amino acids.

As both recombinant (r)SmLy6A and B were expressed, purified and dialysed using the same protocols, we list here the common set of methods used but only refer to rSmLy6A production to avoid confusion. The pET30a/SmLy6A plasmid was transformed into chemically competent *E*. *coli* BL21 star (DE3) cells (Invitrogen) and expression of rSmLy6A followed the protocols listed in the BL21 star (DE3) manual. Four hours after isopropyl β-D-1-thiogalactopyranoside (IPTG) induction (0.5mM final concentration), bacteria were pelleted, lysed and the resultant insoluble fraction was then used to purify the recombinant protein. The pelleted insoluble fraction containing rSmLy6A was first re-suspended in a wash buffer consisting of 500mM NaCl, 50mM Tris-HCl, 10mM EDTA, 0.5% Triton-X-100, pH 8. After one hour on a rocking platform, the sample was pelleted and a second wash (wash buffer + 3M urea) was performed using the same procedure as above. After these wash steps, purification of rSmLy6A was performed under denaturing conditions using Ni-NTA agarose beads (Qiagen) according to the manufacturer’s instructions. The resultant purified protein had urea removed by stepwise dialysis against ≥20 vols of 100mM NaH_2_PO_4_, 10mM Tris-HCl, pH 6.3 buffer containing 6M, 3 M, 1 M, then two buffer changes without urea. Each dialysis step was performed for a minimum of two hours. The final sample was clarified by centrifugation at 21, 000 g for 15 min at 4°C.

The identity of rSmLy6A and rSmLy6B as the sole purified product was confirmed by in-gel trypsin digestion [39], followed by mass spectrometric analysis of extracted peptides (LC-MS/MS for rSmLy6A; protocol as listed in [39], MALDI-TOF for rSmLy6B; protocol as listed in [40]) and subsequent sequence identification by a Mascot (version 2.2.1; Matrix Science) database search of the *S*. *mansoni* predicted proteins from genome assembly 4.0. Recombinant SmTAL1 was expressed and purified as previously described [41].

### Anti-sera production and western blots

Antisera against rSmLy6A or rSmLy6B emulsified with Alum adjuvant (Alu-Gel-S, Serva, Germany) were raised in male 8-week-old Fischer rats (Charles River). Intraperitoneal injections of rSmLy6A or rSmLy6B were performed on days 0 (50μg), 21 (30μg) and 35 (30μg) with terminal bleeds performed at day 56.

Soluble parasite proteins were prepared from mixed-sex 24-hr schistosomula and 7-week adult worms by the addition of an extraction buffer (5mM Tris, 400mM KCl, 1% Triton-X100, 10mM EDTA, 1% Protease Inhibitors (Sigma-Aldrich) pH 9.2), sonication, two freeze/thaw cycles and centrifugation at 17, 000 x g for 15 minutes. Soluble parasite proteins were separated using SDS–PAGE, transferred to a polyvinylidene difluoride (PVDF) membrane and then probed as previously described [42]. The polyclonal anti-rSmLy6A rat antiserum was used at a 1:5,000 dilution while the polyclonal anti-rSmLy6B rat antiserum was used at a 1:2,000 dilution. Rabbit anti-rat IgG peroxidase-conjugated secondary antibody (Sigma-Aldrich) (1:5000) and chemiluminescence (Chemiluminescent Peroxidase Substrate-3) were used according to the manufacturer’s recommendation (Sigma-Aldrich). Western blots of rSmLy6A and rSmLy6B were performed using the same method and reagents as listed above with the replacement of both primary antiserum and secondary antibody incubations with a single HisProbe-HRP (Pierce) incubation at 1:4,000 dilution.

### Human study population

The study cohort included inhabitants of Musoli, a fishing community on Lake Victoria, Uganda, Africa. Descriptions of cohort selection, quantitative parasitology and treatment regimes for this study are communicated in a previous publication [43]. In this report we focused on 216 members of the cohort who were under 60 years of age and who donated blood before and 9 weeks after they received praziquantel (PZQ).

### Human sera ELISA

IgE, IgG_1_ and IgG_4_ levels for recombinant Tegumental Allergen-Like protein 1 (rSmTAL1) were measured by isotype-specific ELISA as described previously [43]. IgE, IgG_1_ and IgG_4_ levels against rSmLy6A and rSmLy6B were measured using a similar protocol with the following modifications. Coating concentrations were 6.25 and 9.6μg/ml for rSmLy6A and rSmLy6B respectively. To measure IgE, plasma was diluted 1:20 with 10% (v/v) fetal calf serum (FCS) and to measure IgG_1_ or IgG_4_ plasma was diluted 1:200 with 1% (v/v) FCS. Wells were incubated overnight at 4°C, washed and then incubated for 4 h with 0.5 μg/ml biotinylated mouse anti-human IgE (Clone G7-18, Pharmingen), biotinylated mouse anti-human IgG_1_ (Clone G17-7, Pharmingen) or biotinylated mouse anti-human IgG_4_ (Clone JDC-14, Pharmingen), followed by 1:3000 streptavidin/biotinylated-HRP complex (Mast Group Ltd.). The assay was then developed with o-phenylenediamine substrate solution (Sigma) and stopped with 2 M sulphuric acid. Standard curves were generated by control immunoglobulins including IgE (Calbiochem), IgG_1_ (Sigma) or IgG_4_ (Sigma) as appropriate. Plasma samples from 26 uninfected European/North American donors were included in each assay.

### Human serology analysis and statistics

Age was divided into the following groups to account for the non-linear relationship between infection intensity and age: 7–9yrs (n = 39), 10–13yrs (n = 41), 14–23yrs (n = 42), 24–32yrs (n = 44), 38–50yrs (n = 45). Pre-treatment antibody responses were classed as a binomial variable: "responders" and "non-responders". Responders were individuals with Ab response greater than the mean + 3 standard deviations of the response of a plasma panel donated by European/North American individuals. Pre-treatment and post-treatment antibodies levels of positive pre-treatment responders to recombinant proteins were compared using a Wilcoxon signed-rank test.

## Results

As Ly6 proteins share little sequence identity [16,18], we performed a systematic search of the *S*. *mansoni* genome using position-specific iterated BLAST search (PSI-BLAST; [30]). First iteration searches were performed using five representative Ly6 proteins (HsCD59, HsuPAR, DmBoudin, DmRtv and CeODR-2) as query sequences. Sequences for use in subsequent iterations were selected by identifying regions between 55–115aa in length containing 10 cysteines, where Cys^1^ and Cys^2^ were separated by any two residues (C^1^-X-X-C^2^) and Cys^10^ was followed by an Asn residue (C^10^N) (see Materials and Methods for detailed description of PSI-BLAST parameters). This comprehensive search of the *S*. *mansoni* genome, using canonical characteristics of the Ly6 family, resulted in the identification of eleven *S*. *mansoni* Ly6 members named SmLy6A-K (see Table 1). All eleven SmLy6 transcript sequences were verified by PCR (PCR primers listed in S1 Table) from parasite cDNA, with complete open reading frames (ORF) verified for nine transcripts (SmLy6A-H and J) and partial ORFs confirmed for two (SmLy6I, K). Of these eleven Ly6 members, eight have been previously reported—SmLy6A and SmLy6B in a tegumental proteomic study ([11]; SmCD59a and b respectively), SmLy6D, which is the tegumental vaccine candidate Sm29 [44,45] and seven SmLy6 members (SmLy6A-F, I and K) in a recent publication also characterizing this family [27]. SmLy6G, Ly6H and Ly6J represent novel *S*. *mansoni* family members.

Examination of SmLy6 gene positions within the *S*. *mansoni* genome finds seven of the eleven family members present on the chromosome 1 with four present in a 41,000bp contiguous cluster (SmLy6I, SmLy6K, SmLy6F and SmLy6C; Table 1). Putative orthologues for SmLy6 genes were also identified in the *S*. *haematobium* [12] and *S*. *japonicum* [46] genomes using BLAST searches against gene predictions, with SmLy6A, SmLy6B, SmLy6H, SmLy6J and SmLy6K possessing particularly high levels of sequence identity to *S*. *haematobium* orthologs (≥80% at the protein sequence level; Table 1).

### SmLy6 proteins possess features consistent with Ly6 family members

**Fig 1:**
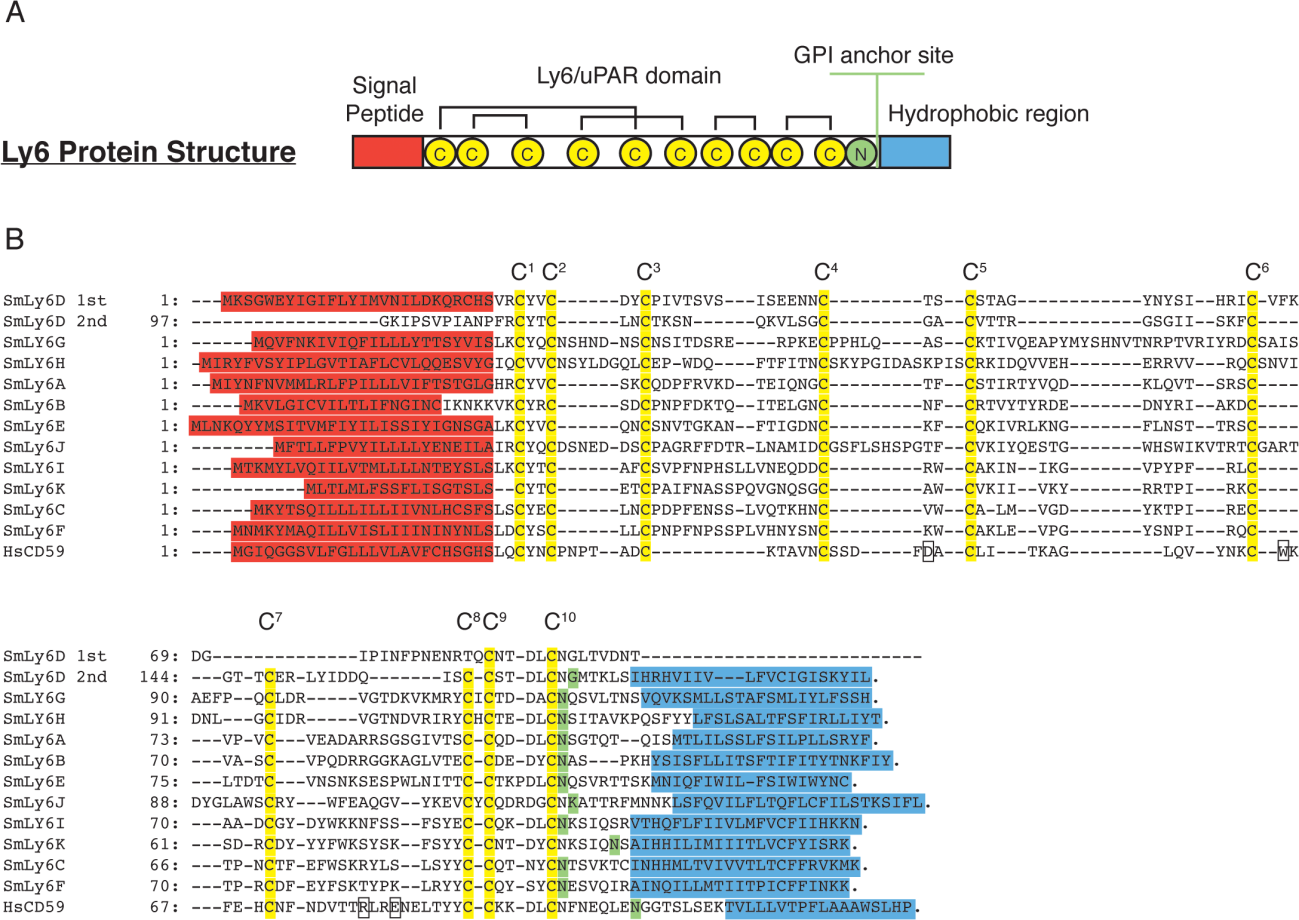
SmLy6 members demonstrate feature conservation characteristic of the uPAR/Ly6 superfamily. PSI-BLAST querying was used to identify eleven Ly6 family members in the *S*. *mansoni* genome (v 5.0). A) Diagrammatic representation of protein features characteristic of a single domain Ly6 protein. B) MUSCLE alignment of the deduced full-length amino acid sequences of SmLy6A—K sequences (SmLy6D sequence is split into SmLy6D 1^st^ and 2^nd^ domains). Predicted signal peptide sequences are highlighted in red. Conserved cysteines are shaded in yellow and labelled C^1^ to C^10^. Predicted GPI anchor sites are shaded in green and hydrophobic regions are highlighted in blue. The four amino acids shown to be essential in HsCD59 function [47] are indicated on the sequence by boxes. The amino acid numbering (indicated at left of alignment) begins at the initiating M of each protein.

Alignment of the amino acid sequences encoded by SmLy6A-K revealed low levels of identity (average 28%) across the putative uPAR/Ly6 domains, but did show conservation of the ten canonical cysteine residues across all SmLy6 proteins (Fig 1; HsCD59 sequence included as a representative Ly6 protein). A single uPAR/Ly6 domain was identified in all but one of the SmLy6 protein, with SmLy6D containing two. Examination of the two SmLy6D uPAR/Ly6 domains finds that the two cysteines (C^7^ and C^8^) which form the fifth disulphide bond in the uPAR/Ly6 domain are absent in the 1^st^ domain but present in the second (Fig 1; yellow residues). For all eleven family members, the spacing between the C-terminal cysteines—C^8^ to C^9^ (0 or 1 residues) and C9 to C^10^ (3–5 residues)—is consistent with the inclusion of these proteins into the uPAR/Ly6 superfamily (Fig 1).

All eleven SmLy6 proteins also contained a predicted hydrophobic signal peptide sequence (SignalP program; [33]) as well as a C-terminal hydrophobic region (TMpred program; [34]), both of which are characteristic of Ly6 proteins (Fig 1; signal peptide in red, hydrophobic region in blue). Predicted GPI-anchor sites (BIGPI server; [35]) were found to be highly conserved in all SmLy6 proteins with eight of the sequences (SmLy6A, B, C, E, F, G, H, I) most likely to be modified at the conserved N residue, while the GPI-attachment sites for SmLy6D, J and K located nearby (Fig 1; green residues). Examination of the SmLy6 amino acid sequences fails to find conservation of the four residues (Fig 1, boxes) known to be important in HsCD59 function [47] in any of the SmLy6 family members.

### SmLy6 possess tertiary structure consistent with Ly6 family members

**Fig 2:**
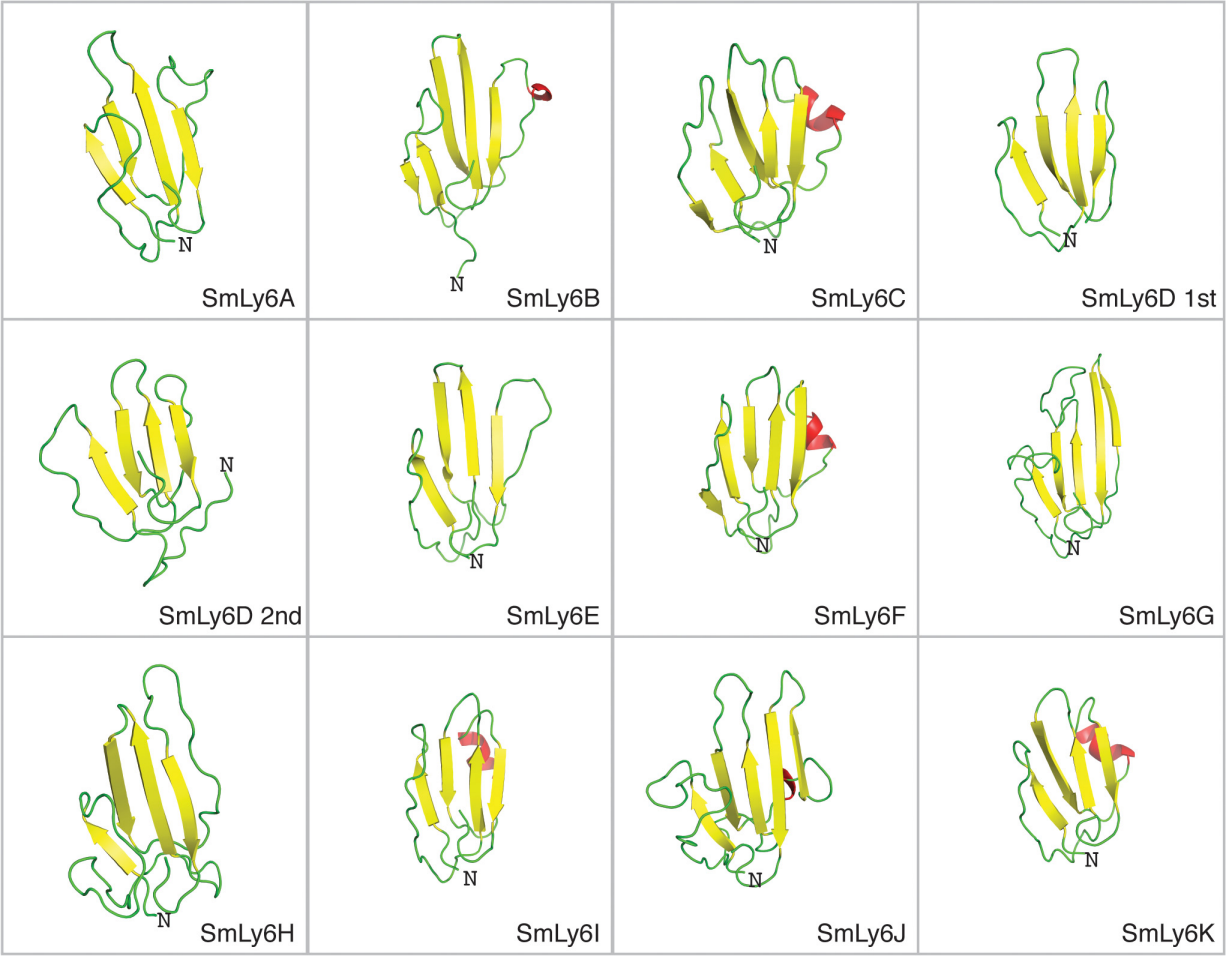
Structural models of SmLy6 sequences reveal common “three-fingered fold” tertiary structures. SmLy6 tertiary structures were *ab initio* modelled using Rosetta software according to the Materials and Methods. From top to bottom, left to right ribbon representation of SmLy6A to SmLy6K where the two Ly6 domains of SmLy6D were modeled independently (SmLy6D 1^st^ and SmLy6D 2^nd^). Beta strand, alpha helices and loops depicted in yellow, red and green respectively. Structural rendering generated using PyMOL.

To explore the tertiary structural characteristics of the mature SmLy6 proteins (signal peptide and residues C-terminal to the GPI anchor removed), *ab initio* modeling was performed (Fig 2) as homology modeling is an unsuitable technique for proteins with limited sequence similarity to homologs [48]. Importantly, all eleven SmLy6 structure models produced by these analyses possessed a conserved core fold, consisting of four beta strands (yellow arrows) forming a beta sheet (Fig 2). These four beta strands (e.g. SmLy6A) match the characteristic ‘three-fingered’ fold of the Ly6 family members such as HsCD59 [19].

### Expression profiles and recombinant production of SmLy6A and B

**Fig 3:**
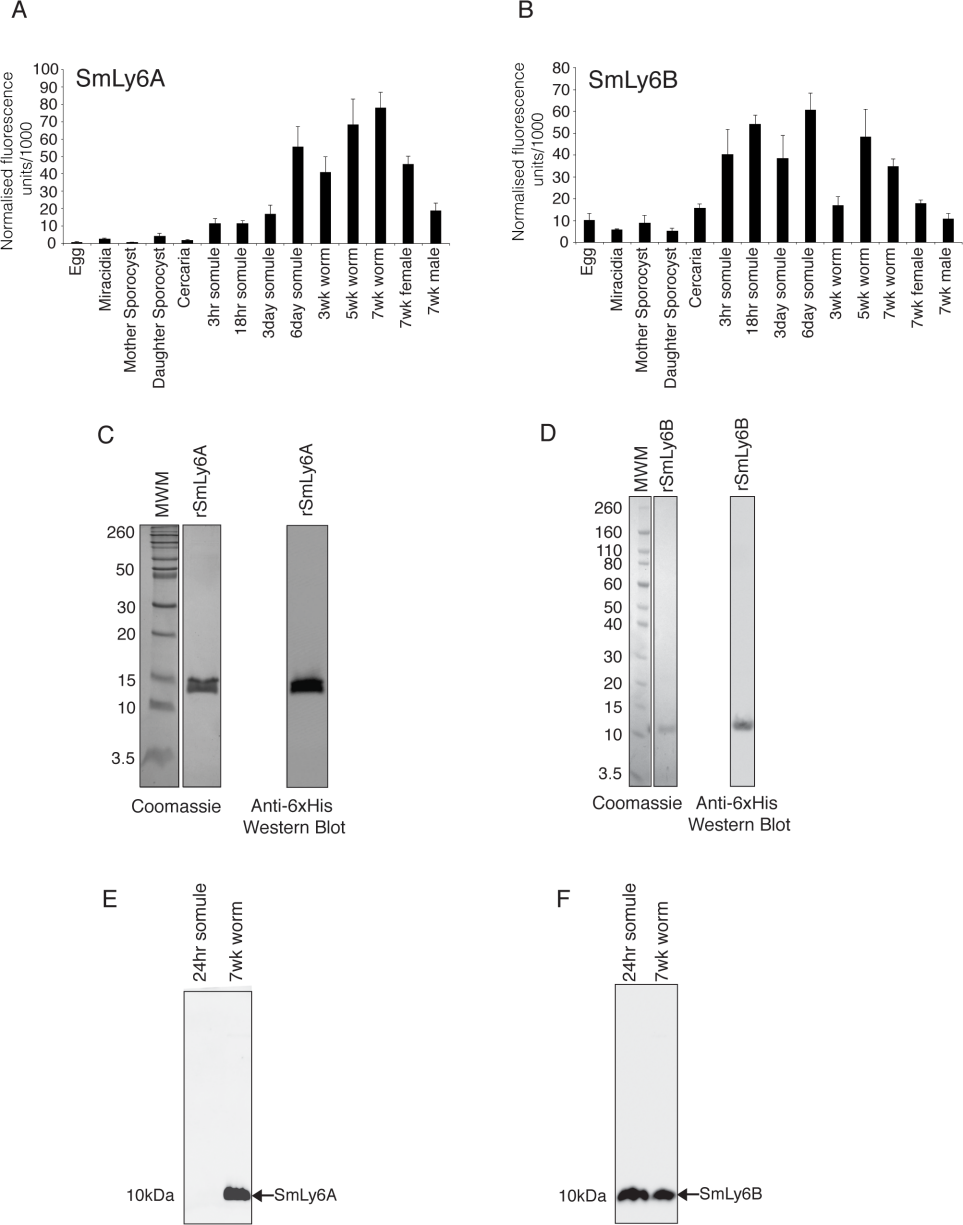
SmLy6A and SmLy6B are both abundantly expressed in adult schistosomes but only SmLy6B is detectable in schistosomula. SmLy6A and SmLy6B mRNA and protein abundances were elucidated as described in the Materials and Methods. Transcription profiles of SmLy6A (A) and SmLy6B (B) were derived from the *S*. *mansoni* lifecycle microarray data available via Array express [10] under the experimental accession number E-MEXP-2094. Values are mean normalized fluorescence units ± sem (standard error of the mean). Recombinant SmLy6A (C) and SmLy6B (D) were cloned into a modified pET30a vector (Novagen), expressed using BL21 star cell line (Invitrogen) and purified from inclusion bodies using Ni-NTA affinity columns (Qiagen). (E) Anti-rSmLy6A polyclonal rat antisera recognises a ~10kDa native protein found within soluble extracts derived from mixed-sex 7-week old worms. (F) Anti-rSmLy6A polyclonal rat antisera recognises a ~10kDa native SmLy6B from 24 schistosomula and 7 week worms. Pre-bleed rat serum did not recognise either protein.

Using information available from a DNA microarray database [10], the mRNA abundance for 8 of the 11 SmLy6 genes (SmLy6A-D and F-I) across 14 different schistosome life-stages was deduced (S1 Fig). The results for SmLy6A (Fig 3A) and SmLy6B (Fig 3B) showed a similar trend (low/no expression in egg/snail-residing parasite stages, but rising abundance within mammalian-residing parasite stages). However, SmLy6B was more abundantly transcribed (~ 2–4X) in the earlier schistosomula stages analysed (3 hr—3 day) when compared to SmLy6A.

Recombinant SmLy6A and B were expressed in *E*. *coli* cells (BL21 star), purified via the C-terminal 6xHis tag under denaturing conditions using Ni-NTA (nickel-nitrilotriacetic acid) affinity chromatography and sequenced to confirm identity (rSmLy6A - Fig 3C, rSmLy6B - Fig 3D). Rat anti-sera raised against both rSmLy6A and rSmLy6B detected native proteins in soluble worm antigen preparations of the correct approximate molecular mass for both native SmLy6A (8.03kDa; Fig 3E) and SmLy6B (8.85kDa; Fig 3F). Anti-rSmLy6B also recognized an appropriate molecular mass protein corresponding to native SmLy6B in soluble 24-hr schistosomula protein extracts (Fig 3F).

### rSmLy6A and rSmLy6B are recognized by infected humans

**Fig 4:**
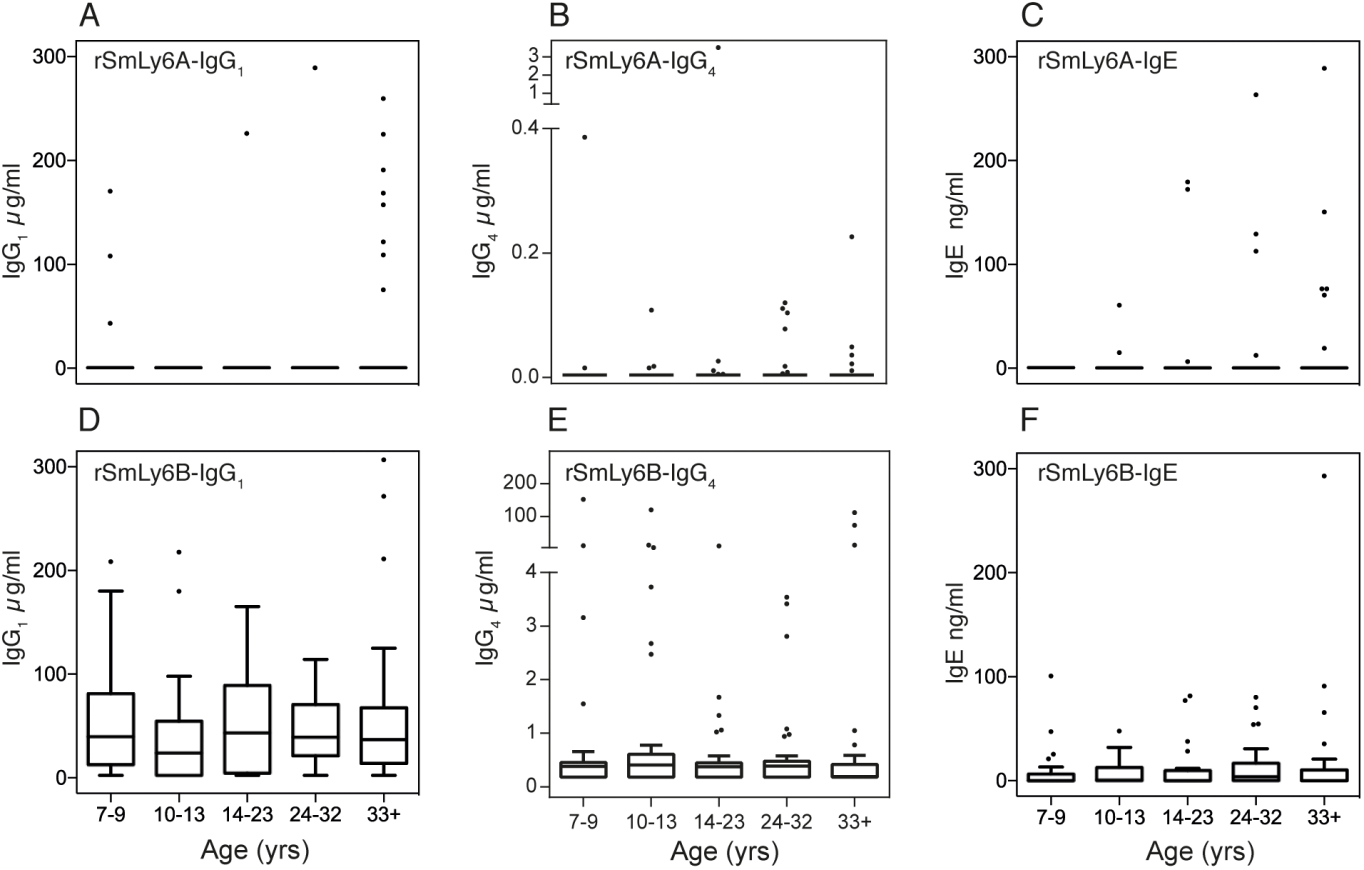
SmLy6A and SmLy6B are differentially recognised in a cohort of *S*. *mansoni* infected males from a Ugandan fishing community. Recombinant SmLy6A and SmLy6B were used in ELISA to measure antigen-specific IgG_1_ (SmLy6A—Panel A, SmLy6B—Panel D), IgG_4_ (SmLy6A—Panel B and SmLy6B—Panel E) and IgE (SmLy6A—Panel C and SmLy6B—Panel F) responses in the plasma of 216 males infected with *S*. *mansoni* prior to praziquantel treatment. The cohort is divided into 5 age groups, 7–9 (n = 36), 10–13 (n = 37), 14–23 (n = 41), 24–32 (n = 42) and 33+ (n = 60) and the antibody levels of each group is presented as box plots.

Antibody responses against rSmLy6A and rSmLy6B were measured in the plasma of a *S*. *mansoni* infected male (aged 7–70 yrs) cohort [43] from a high transmission area in Uganda at two time-points—before and 9 weeks after PZQ treatment (Fig 4). To assess the age profiles of the responses, anti-rSmLy6A and rSmLy6B IgG_1_, IgG_4_ and IgE levels were plotted for five age groups (7–9, 10–13, 14–23, 24–32 and 33+ years) and positive responders (seropositive) were defined as those individuals exhibiting mean anti-rSmLy6 titers above the mean level observed from uninfected European/North American samples + 3x standard deviation (Fig 4).

Before PZQ treatment (Fig 4) detectable IgG_1_ responses to SmLy6A were rare, present in only 7% of the cohort (16 individuals, Fig 4A). In contrast, a sizable percentage (25%) of the cohort contained seropositive rSmLy6B IgG_1_ levels before treatment (Fig 4D). No association was observed between infection intensity and rSmLy6A or rSmLy6B IgG1 responses (S2 Table). Similarly, pre-treatment IgG_4_ responses in the cohort also showed that responses to rSmLy6B were more abundant (2.63μg/ml geometric mean) and more common (21%, Fig 4E) than rSmLy6A—IgG_4_ responses (0.04μg/ml geometric mean, 10% of the cohort, Fig 4B). Finally, IgE responses to rSmLy6A and rSmLy6B (Fig 4C and 4F) were rarer but detectable in some individuals (9 and 4% respectively).

### IgG_1_ responses to SmLy6A and B drop after treatment in infected humans

**Fig 5:**
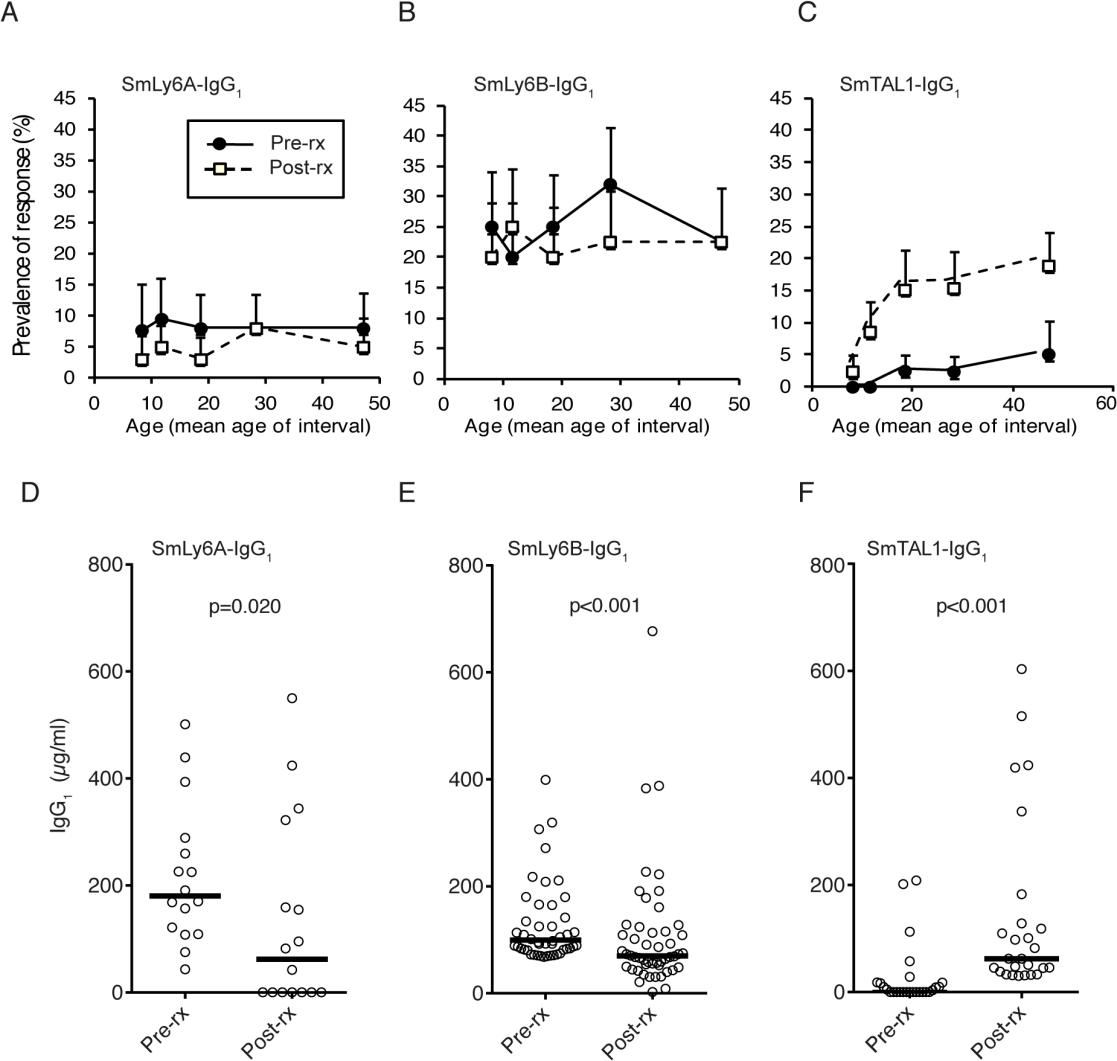
Praziquantel treatment does not alter SmLy6A and SmLy6B IgG_1_ age distribution profiles, but does decrease antigen-specific IgG_1_ reactivity. SmLy6A-, SmLy6B- and SmTAL1-specific IgG_1_ were measured before and 9 weeks after praziquantel treatment in a cohort of infected males (n = 216). A) Age–associated profiles of SmLy6A-, B) SmLy6B-, and C) SmTAL1-specific IgG_1_ responses pre- and post-praziquantel treatment. (D) Effect of praziquantel treatment on human IgG_1_ responses to SmLy6A, (E) SmLy6B and (F) SmTAL1. IgG_1_ levels for those individuals producing a detectable response (>mean+3xSD uninfected controls) to SmLy6A (n = 16), SmLy6B (n = 50) or SmTAL1 (n = 26) before treatment are shown and the median value is represented by a horizontal bar. *P*-values were calculated using a Wilcoxon signed-rank test comparing pre-treatment and post-treatment antibody levels.

To further analyse the endemic human antibody responses to rSmLy6A and rSmLy6B in this infected cohort, pre- and post-PZQ treatment antibody levels were measured. In addition to rSmLy6A and rSmLy6B, antibody levels against rSmTAL1 (a non-surface tegumental antigen previously known as Sm22.6), were compared to assess whether protein localization within the tegument may influence antibody responses. For IgG_1_ responses, anti-rSmLy6A and rSmLy6B showed no age-associated profile either pre- or post-PZQ treatment, with prevalence in all age groups comparable (Fig 5A and 5B). In contrast, SmTAL1-IgG_1_ responses showed clear age-associated profiles of increasing prevalence with age in both pre- and post-PZQ treatment (Fig 5C). This finding was also observed in the IgG_4_ and IgE responses where no age-associated relationships were observed pre- or post-PZQ treatment to SmLy6A and B, when compared to a positive association detected for rSmTAL1 (see S2 Table).

In those males that were seropositive pre-treatment for rSmLy6B, levels of rSmLy6B-IgG_1_ decreased significantly at 9 weeks post-treatment (*p*<0.001 n = 50; Fig 5E) with a similar trend observed for SmLy6A-IgG_1_ (*p* = 0.02, n = 16; Fig 5D). This is the reverse of the SmTAL1-IgG_1_ response dynamics, where the response increased significantly at 9 weeks post-treatment (*p*<0.001 n = 26; Fig 5F). For IgG_4_, the relatively low levels of rSmLy6A-IgG_4_ did not significantly change between pre-treatment and 9 weeks post-treatment (S2 Fig), but there was a significant drop in rSmLy6B-IgG_4_ (p<0.001, n = 193; S2 Fig). The minimal IgE responses to rSmLy6A and rSmLy6B did not change significantly following treatment (S2 Fig).

## Discussion

Investigating the human immune responses directed against schistosome surface proteins is a logical step in the progression of next-generation vaccine candidates to combat schistosomiasis [49]. Here we have focused our investigation on the Ly6 protein family as previously studies have indicated that three members are found on the schistosome surface tethered by a GPI-anchor (SmLy6A, SmLy6B and SmLy6D; [11]), two are capable of inducing protective immunity in the mouse model (SmLy6B [50] and SmLy6D [44]) and one is recognised during human schistosomiasis (SmLy6D; [45]).

Our genome analysis of this family identifies eleven members (SmLy6A-K), each possessing the characteristic features of Ly6 proteins including a signal peptide, a uPAR/Ly6 domain in a TFPD fold with conserved cysteine spacing, a potential GPI-anchor site and a C-terminal hydrophobic region (see Table 1 and Fig 1). Positioning in the genome suggests recent gene duplication events, but the lack of highly similar (>95% identity) orthologues in the *S*. *japonicum* and *S*. *haematobium* genomes likely reflects rapid divergence of these genes as observed in Ly6 genes found in other species [14,16,18]. With the identification of SmLy6A, B, C and D in the adult worm tegument [11,26] and Ly6 family members also present in the tegument of *Fasciola hepatica* adults [51], it is clear that a sub-set of these schistosome proteins function at the host/parasite interface. Farias *et al*. have clearly demonstrated that SmLy6 members do not function as CD59 orthologs [27], but whether these schistosome proteins interact with other host molecules or help maintain the tegumental barrier (potentially related to the septate junction function of *Dm-*boudin; [18]) is currently unknown.

The nomenclature used to describe these proteins within the schistosome scientific community is currently inconsistent with SmCD59a, SmCD59b, Dif-5 and Sm29 all previously being used (see Table 1). Additionally, during the completion of this study, a report by Farias *et al*. described a sub-set of this Ly6 family in which the authors explicitly named them SmCD59.1-.7. [27]. We have chosen to use the name SmLy6 for these proteins in our study as: (a) sequence analysis does not support greater similarity to CD59 than other specific Ly6 members, (b) functional orthology to the CD59 protein was also comprehensively disproved by Farias *et al*. [27] and (c) the most commonly used designation for this class of protein is Ly6 (signal peptide-containing, GPI-anchored proteins with a TFPD fold) [21,23]. The discrepancy between the number of Ly6 members identified in this study (eleven members) and that described by Farias *et al*. (seven members) reflects the different search methods used. Our PSI-BLAST methodology has been used previously to identify the *D*. *melanogaster* Ly6 family and is particularly powerful where sequence similarity is low overall, but specific protein motifs are conserved [18]. In this way we have successfully extended the family membership by over 50% from the search performed by Farias *et al*., however it remains possible that other divergent members were not identified in this study (specifically smp_202630 and smp_064430 as they bear similarities to the family such as the presence of the C^1^-X-X-C^2^ and C^10^N motif). Perhaps the most surprising new member of the Ly6 family is the potential vaccine candidate Sm29 (Table 1; SmLy6D).

Sm29 was first described in 2006 as a tegumental protein of unknown function and sharing no sequence similarity to any known protein family [45]. However, our PSI-BLAST analysis of the *S*. *mansoni* genome demonstrated that Sm29 contained all major Ly6 features (Fig 1; SmLy6D) with *ab initio* modeling clearly showing the TFPD fold characteristic of the Ly6 family (Fig 2; SmLy6D). The presence of two uPAR/Ly6 domains in SmLy6D makes it unique in the SmLy6 family, as all the other members have only one domain (Fig 1). This particular feature is similar to human uPAR protein, which contains three tandem uPAR/Ly6 domains. SmLy6D is also comparable to uPAR by lacking the C7-C8 disulphide bond within the first uPAR/Ly6 domain (Fig 1). uPAR’s binding with uPA has been shown by site-directed mutagenesis to be coordinated by four residues present in the first uPAR/Ly6 domain (Arg53, Leu55, Tyr57, and Leu66) between C6 and C9 [52]. Intriguingly, in the same region of its second uPAR/Ly6 domain, SmLy6D possesses an Arg, Leu, Tyr, Ile tetrapeptide sequence (Fig 1). Whether these four residues truly form the functional residues for Sm29/SmLy6D is beyond the scope of this study, however, we believe it warrants further investigation.

Our co-measurement of SmTAL1 and SmLy6A/SmLy6B serological responses in the same infection cohort provided an opportunity to compare antibody isotype responses to proteins found on (SmLy6A/SmLy6B; [11,27]) or below (SmTAL1; [43]) the schistosome surface. Here, we observed clear differences in both the age-associated profiles as well as the predominant serotypes between SmTAL1 and the studied SmLy6 proteins (Fig 5).

As shown in Fitzsimmons *et al*., almost half of this cohort was seropositive for SmTAL1-IgE and SmTAL1-IgG_4_ (45% in both cases), with fewer individuals seropositive for SmTAL1-IgG_1_ (12%; [43]). These strongly IgE/IgG_4_-dominated serotype responses have also been observed against other non-surface (cryptic) *S*. *mansoni* proteins such as Cathepsin B1 (SmCB1—a gut associated peptidase; [53]) and Glutathione-S-transferase (Sm28-GST—a parenchymal protein [54]). The IgE/IgG_4_ dominated response against SmTAL1 and other non-surface proteins [55] contrasts with the results observed for SmLy6A and SmLy6B, which comprise mainly of IgG_1_ antibodies, with lower levels of IgG_4_ and IgE (Fig 4). These findings are consistent with other vaccine candidates found on or near the surface of the parasite [56] such as Tetraspanin-2 (SmTSP-2) [57], Sm23 [58] and Glyceraldehyde 3-Phosphate Dehydrogenase (SG3PDH) [59,60]. Amongst the SmLy6 family, serological responses within a small Brazilian cohort of schistosome-infected humans against the surface-exposed SmLy6D (Sm29) have previously been investigated [45]. Here, the authors identified high anti-SmLy6D IgG_1_ titres, with no anti-SmLy6D IgG_4_ or IgE component detected in infected individuals. This is broadly similar to our study. However, we have also identified a sizable percentage of low titre IgG_4_ responders and a few detectable IgE responses to both SmLy6A and SmLy6B (Fig 4). These data indicate that single Ly6 (SmLy6A and SmLy6B) versus multiple Ly6 (SmLy6D)-domain containing family members can induce different immunological responses in endemic populations. Whether these isotypic differences between single and double domain Ly6 family members are found in other schistosome-infected cohorts, are influenced by differential SmLy6 protein abundance or whether other single Ly6-domain containing family members also elicit an IgG_4_/IgE response is currently unknown, but requires further investigation for future immunoprophylaxis consideration of the SmLy6 family.

SmLy6A and SmLy6B serological responses also differ from SmTAL1 in terms of age-association profiles (Fig 5A–5C) and post-treatment PZQ effects (Fig 5D–5F). Collectively, and regardless of prevalence (SmLy6B – 25% > SmLy6A – 7%), anti-SmLy6 IgG_1_ immune responses did not significantly change with age and dropped upon PZQ treatment (Fig 5A and 5B) in the studied population. In contrast, IgG_1_ responses against SmTAL1 demonstrated a positive association with age group analysed and were boosted by chemotherapy (Fig 5C). These data suggest that, while sub-surface SmTAL1 only becomes exposed to host antibody responses upon natural or drug mediated parasite death [43], SmLy6 proteins (SmLy6B>SmLy6A) are more likely to be recognised in endemic populations due to their surface localisation in live parasites. However, the anti-Ly6 antibody response may also be influenced by the difference in surface exposure of these antigens during parasite development as recently demonstrated for SmLy6A (SmCD59a) [61]. Here, Reimers *et al*. illustrated that SmLy6A was not abundantly found on the surface of both 7 and 14 day schistosomula (despite being detectable in soluble protein extracts), indicating that this particular Ly6 member is inaccessible to antibodies unless damaged [61]. The prevalence/magnitude of anti-SmLy6 IgG_1_ responses may, therefore, be linked to antigen abundance (surface or sub-surface) in the schistosomula stages (SmLy6B is expressed in earlier lifecycle stages than SmLy6A; Fig 3) and/or low-level cross reactivity (SmLy6A and SmLy6B share 42% sequence identity at the amino acid level). Support for cross-reactivity is present in this study, with all of the SmLy6A IgG_1_ responders (n = 16) also IgG_1_ responders to SmLy6B. However, between isotypes, there is minimal overlap in individuals responding with no individuals positive for all three isotypes tested (IgG1, IgG4 and IgE) against SmLy6A and only three of the eighty-five individuals positive for all three antibody isotypes against SmLy6B.

In this study, we have comprehensively characterized the *S*. *mansoni* Ly6 family, finding eleven members (three novel proteins and the new classification of Sm29 as a Ly6 family member) that possess protein features and tertiary fold structures distinctive of membrane-associated localisation. Comparative isotypic, age-association and post-PZQ treatment antibody responses to two representative members (SmLy6A and SmLy6B) in a cohort of *S*. *mansoni* infected males from a Ugandan fishing community strongly support their membrane association at the adult schistosome surface (supporting [11] and [27]), when viewed in light of the immunological results generated for sub-surface SmTAL1. This unique immunological comparison within the same cohort provides clear evidence for the plasticity of the human immune response to different parasite proteins depending on lifecycle expression and tegumental localisation. We, therefore, contend that both temporal and spatial expression of SmLy6 members are equally important in initiating immune responses; a factor that may have relevance in the progression of this family as next generation immunoprophylactic candidates.

## Supporting Information

S1 Table Primers used for SmLy6 family member PCR amplification. Gene product of interest, sequences of the primer pair and the annealing temperature used are listed. Annealing temperatures refer to those used in PCR reaction with Phusion polymerase.
(DOCX)

S2 Table Analysis of serological responses to SmLy6A and B related to age and infection intensity. Age was divided into the following groups to account for the non-linear relationship between infection intensity and age: 7–9yrs (n = 39), 10–13yrs (n = 41), 14–23yrs (n = 42), 24–32yrs (n = 44), 38–50yrs (n = 45). Pre-treatment antibody responses were classed as a binomial variable: "responders" and "non-responders". Responders were individuals with antibody response greater than the mean + 3 standard deviations of the response of a plasma panel donated by European/North American individuals. Antibody responses were analysed by logistic regression, controlling for age-group and infection intensity. IgE models could not be computed due to some age groups having n value of zero.
(DOCX)

S1 Fig Transcription profiles of SmLy6 family members reveal association with mammalian parasitism. Profiles from the *S*. *mansoni* lifecycle DNA microarray data available via Array express [10] under the experimental accession number E-MEXP-2094. Values are mean normalized fluorescence units ± sem.
(TIF)

S2 Fig Praziquantel treatment effect on SmLy6A and SmLy6B IgG_4_ and IgE reactivity. SmLy6A- and SmLy6B-specific IgG_4_ and IgE were measured before and 9 weeks after praziquantel treatment in a cohort of infected males. Pre and post-praziquantel treatment IgG4 and IgE antibody responses to SmLy6A and SmLy6B, including median value and interquartile range. Statistical analysis was performed using the Wilcoxon Signed Rank test (significance level P<0.05, n = 193).
(TIF)

## References

[1] Crompton DW (1999) How much human helminthiasis is there in the world? J Parasitol 85: 397–403. 10386428

[2] King CH, Dangerfield-Cha M (2008) The unacknowledged impact of chronic schistosomiasis. Chronic Illn 4: 65–79. 10.1177/1742395307084407 18322031

[3] Colley DG, Bustinduy AL, Secor WE, King CH (2014) Human schistosomiasis. Lancet 383: 2253–2264. 10.1016/S0140-6736(13)61949-2 24698483 PMC4672382

[4] Ismail M, Metwally A, Farghaly A, Bruce J, Tao LF, et al (1996) Characterization of isolates of *Schistosoma mansoni* from Egyptian villagers that tolerate high doses of praziquantel. Am J Trop Med Hyg 55: 214–218. 8780463 10.4269/ajtmh.1996.55.214

[5] Bergquist NR (1998) Schistosomiasis vaccine development: progress and prospects. Mem Inst Oswaldo Cruz 93 Suppl 1: 95–101. 9921329 10.1590/s0074-02761998000700013

[6] Berriman M, Haas BJ, LoVerde PT, Wilson RA, Dillon GP, et al (2009) The genome of the blood fluke *Schistosoma mansoni* . Nature 460: 352–358. 10.1038/nature08160 19606141 PMC2756445

[7] Hansell E, Braschi S, Medzihradszky KF, Sajid M, Debnath M, et al (2008) Proteomic analysis of skin invasion by blood fluke larvae. PLoS Negl Trop Dis 2: e262 10.1371/journal.pntd.0000262 18629379 PMC2467291

[8] Protasio AV, Tsai IJ, Babbage A, Nichol S, Hunt M, et al (2012) A systematically improved high quality genome and transcriptome of the human blood fluke *Schistosoma mansoni* . PLoS Negl Trop Dis 6: e1455 10.1371/journal.pntd.0001455 22253936 PMC3254664

[9] Mulvenna J, Moertel L, Jones MK, Nawaratna S, Lovas EM, et al (2010) Exposed proteins of the *Schistosoma japonicum* tegument. Int J Parasitol 40: 543–554. 10.1016/j.ijpara.2009.10.002 19853607

[10] Fitzpatrick JM, Peak E, Perally S, Chalmers IW, Barrett J, et al (2009) Anti-schistosomal intervention targets identified by lifecycle transcriptomic analyses. PLoS Negl Trop Dis 3: e543 10.1371/journal.pntd.0000543 19885392 PMC2764848

[11] Castro-Borges W, Dowle A, Curwen RS, Thomas-Oates J, Wilson RA (2011) Enzymatic shaving of the tegument surface of live schistosomes for proteomic analysis: a rational approach to select vaccine candidates. PLoS Negl Trop Dis 5: e993 10.1371/journal.pntd.0000993 21468311 PMC3066142

[12] Young ND, Jex AR, Li B, Liu S, Yang L, et al (2012) Whole-genome sequence of *Schistosoma haematobium* . Nature Genetics 44: 221–225. 10.1038/ng.1065 22246508

[13] Su B, Waneck GL, Flavell RA, Bothwell AL (1991) The glycosyl phosphatidylinositol anchor is critical for Ly-6A/E-mediated T cell activation. J Cell Biol 112: 377–384. 1825084 10.1083/jcb.112.3.377 PMC2288838

[14] Bamezai A (2004) Mouse Ly-6 proteins and their extended family: markers of cell differentiation and regulators of cell signaling. Arch Immunol Ther Exp (Warsz) 52: 255–266. 15467490

[15] Punta M, Coggill PC, Eberhardt RY, Mistry J, Tate J, et al (2012) The Pfam protein families database. Nucleic Acids Res 40: D290–301. 10.1093/nar/gkr1065 22127870 PMC3245129

[16] Galat A (2008) The three-fingered protein domain of the human genome. Cell Mol Life Sci 65: 3481–3493. 10.1007/s00018-008-8473-8 18821057 PMC11131612

[17] Greenwald J, Fischer WH, Vale WW, Choe S (1999) Three-finger toxin fold for the extracellular ligand-binding domain of the type II activin receptor serine kinase. Nat Struct Biol 6: 18–22. 9886286 10.1038/4887

[18] Hijazi A, Masson W, Auge B, Waltzer L, Haenlin M, et al (2009) boudin is required for septate junction organisation in Drosophila and codes for a diffusible protein of the Ly6 superfamily. Development 136: 2199–2209. 10.1242/dev.033845 19502482

[19] Leath KJ, Johnson S, Roversi P, Hughes TR, Smith RA, et al (2007) High-resolution structures of bacterially expressed soluble human CD59. Acta Crystallogr Sect F Struct Biol Cryst Commun 63: 648–652. 17671359 10.1107/S1744309107033477 PMC2335151

[20] Moussian B, Soding J, Schwarz H, Nusslein-Volhard C (2005) Retroactive, a membrane-anchored extracellular protein related to vertebrate snake neurotoxin-like proteins, is required for cuticle organization in the larva of *Drosophila melanogaster* . Dev Dyn 233: 1056–1063. 15844167 10.1002/dvdy.20389

[21] Chou JH, Bargmann CI, Sengupta P (2001) The *Caenorhabditis elegans* odr-2 gene encodes a novel Ly-6-related protein required for olfaction. Genetics 157: 211–224. 11139503 10.1093/genetics/157.1.211 PMC1461492

[22] Garza-Garcia A, Harris R, Esposito D, Gates PB, Driscoll PC (2009) Solution structure and phylogenetics of Prod1, a member of the three-finger protein superfamily implicated in salamander limb regeneration. PLoS One 4: e7123 10.1371/journal.pone.0007123 19771161 PMC2740830

[23] Mallya M, Campbell RD, Aguado B (2006) Characterization of the five novel Ly-6 superfamily members encoded in the MHC, and detection of cells expressing their potential ligands. Protein Sci 15: 2244–2256. 17008713 10.1110/ps.062242606 PMC2242401

[24] Davies A, Simmons DL, Hale G, Harrison RA, Tighe H, et al (1989) CD59, an LY-6-like protein expressed in human lymphoid cells, regulates the action of the complement membrane attack complex on homologous cells. J Exp Med 170: 637–654. 2475570 10.1084/jem.170.3.637 PMC2189447

[25] Roldan AL, Cubellis MV, Masucci MT, Behrendt N, Lund LR, et al (1990) Cloning and expression of the receptor for human urokinase plasminogen activator, a central molecule in cell surface, plasmin dependent proteolysis. Embo J 9: 467–474. 1689240 10.1002/j.1460-2075.1990.tb08132.x PMC551688

[26] van Balkom BW, van Gestel RA, Brouwers JF, Krijgsveld J, Tielens AG, et al (2005) Mass spectrometric analysis of the *Schistosoma mansoni* tegumental sub-proteome. J Proteome Res 4: 958–966. 15952743 10.1021/pr050036w

[27] Farias LP, Krautz-Peterson G, Tararam CA, Araujo-Montoya BO, Fraga TR, et al (2013) On the Three-Finger Protein Domain Fold and CD59-Like Proteins in *Schistosoma mansoni* . PLoS Negl Trop Dis 7: e2482 10.1371/journal.pntd.0002482 24205416 PMC3812095

[28] Lewis FA (1998) Schistosomiasis. Curr Protoc Immunol 28(Suppl): 19.11.11–19.11.28.

[29] Chalmers IW, McArdle AJ, Coulson RM, Wagner MA, Schmid R, et al (2008) Developmentally regulated expression, alternative splicing and distinct sub-groupings in members of the *Schistosoma mansoni* venom allergen-like (SmVAL) gene family. BMC Genomics 9: 89 10.1186/1471-2164-9-89 18294395 PMC2270263

[30] Altschul SF, Madden TL, Schaffer AA, Zhang J, Zhang Z, et al (1997) Gapped BLAST and PSI-BLAST: a new generation of protein database search programs. 25: 3389–3402. 9254694 10.1093/nar/25.17.3389 PMC146917

[31] Pruitt KD, Tatusova T, Maglott DR (2007) NCBI reference sequences (RefSeq): a curated non-redundant sequence database of genomes, transcripts and proteins. Nucleic Acids Res 35: D61–65. 17130148 10.1093/nar/gkl842 PMC1716718

[32] Edgar RC (2004) MUSCLE: multiple sequence alignment with high accuracy and high throughput. Nucleic Acids Res 32: 1792–1797. 15034147 10.1093/nar/gkh340 PMC390337

[33] Bendtsen JD, Nielsen H, von Heijne G, Brunak S (2004) Improved prediction of signal peptides: SignalP 3.0. J Mol Biol 340: 783–795. 15223320 10.1016/j.jmb.2004.05.028

[34] Hofmann KaS, W. (1993) TMBASE—a database of membrane spanning protein segments. Biol Chem Hoppe-Seyler 374: 166.

[35] Eisenhaber B, Bork P, Eisenhaber F (1999) Prediction of potential GPI-modification sites in proprotein sequences. J Mol Biol 292: 741–758. 10497036 10.1006/jmbi.1999.3069

[36] Wynn TA, Eltoum I, Cheever AW, Lewis FA, Gause WC, et al (1993) Analysis of cytokine mRNA expression during primary granuloma formation induced by eggs of *Schistosoma mansoni* . J Immunol 151: 1430–1440. 8335939

[37] Leaver-Fay A, Tyka M, Lewis SM, Lange OF, Thompson J, et al (2011) ROSETTA3: an object-oriented software suite for the simulation and design of macromolecules. Methods Enzymol 487: 545–574. 10.1016/B978-0-12-381270-4.00019-6 21187238 PMC4083816

[38] Raman S, Vernon R, Thompson J, Tyka M, Sadreyev R, et al (2009) Structure prediction for CASP8 with all-atom refinement using Rosetta. Proteins 77 Suppl 9: 89–99. 10.1002/prot.22540 19701941 PMC3688471

[39] de Morree A, Hensbergen PJ, van Haagen HH, Dragan I, Deelder AM, et al (2010) Proteomic analysis of the dysferlin protein complex unveils its importance for sarcolemmal maintenance and integrity. PLoS One 5: e13854 10.1371/journal.pone.0013854 21079765 PMC2974636

[40] Meevissen MH, Balog CI, Koeleman CA, Doenhoff MJ, Schramm G, et al (2011) Targeted glycoproteomic analysis reveals that kappa-5 is a major, uniquely glycosylated component of *Schistosoma mansoni* egg antigens. Mol Cell Proteomics 10: M110 005710 10.1074/mcp.M110.005710 21372247 PMC3098592

[41] Fitzsimmons CM, McBeath R, Joseph S, Jones FM, Walter K, et al (2007) Factors affecting human IgE and IgG responses to allergen-like *Schistosoma mansoni* antigens: Molecular structure and patterns of in vivo exposure. Int Arch Allergy Immunol 142: 40–50. 17019080 10.1159/000095997

[42] Hawn TR, Tom TD, Strand M (1993) Molecular cloning and expression of SmIrV1, a *Schistosoma mansoni* antigen with similarity to calnexin, calreticulin, and OvRal1. J Biol Chem 268: 7692–7698. 8463298

[43] Fitzsimmons CM, Jones FM, Stearn A, Chalmers IW, Hoffmann KF, et al (2012) The *Schistosoma mansoni* tegumental-allergen-like (TAL) protein family: influence of developmental expression on human IgE responses. PLoS Negl Trop Dis 6: e1593 10.1371/journal.pntd.0001593 22509417 PMC3317908

[44] Cardoso FC, Macedo GC, Gava E, Kitten GT, Mati VL, et al (2008) *Schistosoma mansoni* tegument protein Sm29 is able to induce a Th1-type of immune response and protection against parasite infection. PLoS Negl Trop Dis 2: e308 10.1371/journal.pntd.0000308 18827884 PMC2553283

[45] Cardoso FC, Pacifico RN, Mortara RA, Oliveira SC (2006) Human antibody responses of patients living in endemic areas for schistosomiasis to the tegumental protein Sm29 identified through genomic studies. Clin Exp Immunol 144: 382–391. 16734606 10.1111/j.1365-2249.2006.03081.x PMC1941986

[46] Liu F, Zhou Y, Wang Z, G L (2009) The *Schistosoma japonicum* genome reveals features of host-parasite interplay. Nature 460: 345–351. 10.1038/nature08140 19606140 PMC3747554

[47] Bodian DL, Davis SJ, Morgan BP, Rushmere NK (1997) Mutational analysis of the active site and antibody epitopes of the complement-inhibitory glycoprotein, CD59. J Exp Med 185: 507–516. 9053451 10.1084/jem.185.3.507 PMC2196035

[48] Ginalski K (2006) Comparative modeling for protein structure prediction. Curr Opin Struct Biol 16: 172–177. 16510277 10.1016/j.sbi.2006.02.003

[49] Wilson RA (2012) Proteomics at the schistosome-mammalian host interface: any prospects for diagnostics or vaccines? Parasitology 139: 1178–1194. 10.1017/S0031182012000339 22717150

[50] Farias LP, Tararam CA, Miyasato PA, Nishiyama MY Jr., Oliveira KC, et al (2011) Screening the *Schistosoma mansoni* transcriptome for genes differentially expressed in the schistosomulum stage in search for vaccine candidates. Parasitol Res 108: 123–135. 10.1007/s00436-010-2045-1 20852890

[51] Wilson RA, Wright JM, de Castro-Borges W, Parker-Manuel SJ, Dowle AA, et al (2011) Exploring the *Fasciola hepatica* tegument proteome. Int J Parasitol 41: 1347–1359. 10.1016/j.ijpara.2011.08.003 22019596

[52] Gardsvoll H, Dano K, Ploug M (1999) Mapping part of the functional epitope for ligand binding on the receptor for urokinase-type plasminogen activator by site-directed mutagenesis. J Biol Chem 274: 37995–38003. 10608868 10.1074/jbc.274.53.37995

[53] de Oliveira Fraga LA, Lamb EW, Moreno EC, Chatterjee M, Dvorak J, et al (2010) Rapid induction of IgE responses to a worm cysteine protease during murine pre-patent schistosome infection. BMC Immunol 11: 56 10.1186/1471-2172-11-56 21078176 PMC2993659

[54] Auriault C, Gras-Masse H, Pierce RJ, Butterworth AE, Wolowczuk I, et al (1990) Antibody response of *Schistosoma mansoni*-infected human subjects to the recombinant P28 glutathione-S-transferase and to synthetic peptides. J Clin Microbiol 28: 1918–1924. 2121788 10.1128/jcm.28.9.1918-1924.1990 PMC268078

[55] Farnell EJ, Tyagi N, Ryan S, Chalmers IW, Pinot de Moira A, et al (2015) Known Allergen Structures Predict Schistosoma mansoni IgE-Binding Antigens in Human Infection. Front Immunol 6: 26 10.3389/fimmu.2015.00026 25691884 PMC4315118

[56] Braschi S, Wilson RA (2006) Proteins exposed at the adult schistosome surface revealed by biotinylation. Mol Cell Proteomics 5: 347–356. 16269422 10.1074/mcp.M500287-MCP200

[57] Tran MH, Pearson MS, Bethony JM, Smyth DJ, Jones MK, et al (2006) Tetraspanins on the surface of *Schistosoma mansoni* are protective antigens against schistosomiasis. Nat Med 12: 835–840. 16783371 10.1038/nm1430

[58] Ribeiro de Jesus A, Araujo I, Bacellar O, Magalhaes A, Pearce E, et al (2000) Human immune responses to *Schistosoma mansoni* vaccine candidate antigens. Infect Immun 68: 2797–2803. 10768975 10.1128/iai.68.5.2797-2803.2000 PMC97490

[59] El Ridi R, Shoemaker CB, Farouk F, El Sherif NH, Afifi A (2001) Human T- and B-cell responses to *Schistosoma mansoni* recombinant glyceraldehyde 3-phosphate dehydrogenase correlate with resistance to reinfection with *S*. *mansoni* or *Schistosoma haematobium* after chemotherapy. Infect Immun 69: 237–244. 11119511 10.1128/IAI.69.1.237-244.2001 PMC97877

[60] Tallima H, El Ridi R (2008) *Schistosoma mansoni* glyceraldehyde 3-phosphate dehydrogenase is a lung-stage schistosomula surface membrane antigen. Folia Parasitol (Praha) 55: 180–186. 19202676 10.14411/fp.2008.025

[61] Reimers N, Homann A, Hoschler B, Langhans K, Wilson RA, et al (2015) Drug-induced exposure of Schistosoma mansoni antigens SmCD59a and SmKK7. PLoS Negl Trop Dis 9: e0003593 10.1371/journal.pntd.0003593 25774883 PMC4361651

