# Supplementary material for: Human IgG1 Responses to Surface Localised Schistosoma mansoni Ly6 Family Members Drop following Praziquantel Treatment
Source: PLoS Negl Trop Dis. 2015 Jul 6;9(7):e0003920. doi: 10.1371/journal.pntd.0003920 (PMC4492491; doi:10.1371/journal.pntd.0003920)
Supplement: S1 Table — Gene product of interest, sequences of the primer pair and the annealing temperature used are listed. Annealing temperatures refer to those used in PCR reaction with Phusion polymerase. (DOCX) [file pntd.0003920.s001.docx]

| Transcript | Primer sequence | Annealing |
| --- | --- | --- |
| SmLy6A | ATGATCTACAATTTCAATGTAATGATG | 58 |
|  | TTAAAAGTAACGTGATAACAATGGTA |  |
| SmLy6B | ATGAAGTATTGGGAATTTGTGTTATA | 52 |
|  | TCAATATATGAATTTATTAGTATATGTAAT |  |
| SmLy6C | ATGTCTTCTTATCTGATTTCAGGTT | 58 |
|  | TCACTTCATCTTAACTCTGAAGAAAC |  |
| SmLy6E | AATACTACATGAGTATAACTGTAATG | 58 |
|  | TCAGCAGTTATACCATATCCATATAC |  |
| SmLy6F | ATGAATATGAAGTATATGGCACAAA | 54 |
|  | TCACTTTTTATTAATAAAGAAGCAGATA |  |
| SmLy6G | ATGCAAGTGTTCAATAAAATTG | 58 |
|  | GAGTCGCTCAATTTTAATTAATG |  |
| SmLy6H | ATGATTCGCTATTTCGTCAG | 58 |
|  | GATTCAAGAGGGTCATGTGT |  |
| SmLy6I | ATGACTAAGATGTATCTTGTGCAA | 58 |
|  | TCAGTTTTTCTTATGAATAATAAAGCA |  |
| SmLy6J | ATGTTTACACTGTTGTTTCCTG | 59 |
|  | CTATAAAAAAATCGATTTCGTTG |  |
| SmLy6K | TCTGAACATTTTCTTATTGGAGTACGAC | 60 |
|  | GATTTCAGGTACGTCTTTAAGCTGCTA |  |
