# Supplementary material for: Human IgG1 Responses to Surface Localised Schistosoma mansoni Ly6 Family Members Drop following Praziquantel Treatment
Source: PLoS Negl Trop Dis. 2015 Jul 6;9(7):e0003920. doi: 10.1371/journal.pntd.0003920 (PMC4492491; doi:10.1371/journal.pntd.0003920)
Supplement: S2 Table — Age was divided into the following groups to account for the non-linear relationship between infection intensity and age: 7–9yrs (n = 39), 10–13yrs (n = 41), 14–23yrs (n = 42), 24–32yrs (n = 44), 38–50yrs (n = 45). Pre-treatment antibody responses were classed as a binomial variable: "responders" and "non-responders". Responders were individuals with antibody response greater than the mean + 3 standard deviations of the response of a plasma panel donated by European/North American individuals. Antibody responses were analysed by logistic regression, controlling for age-group and infection intensity. IgE models could not be computed due to some age groups having n value of zero. (DOCX) [file pntd.0003920.s002.docx]

| **SmLy6B** | | | | | | |
| --- | --- | --- | --- | --- | --- | --- |
|  | **IgG1** | | **IgG4** | | **IgE** | |
|  | **OR (95% CI)** | **p-value** | **OR (95% CI)** | **p-value** | **OR (95% CI)** | **p-value** |
| 10-13yrs* | 0.49 (0.02, 5.40) | 0.572 | 1.19 (0.18, 9.66) | 0.852 |  | 0.992 |
| 14-23yrs | 1.01 (0.11, 8.91) | 0.992 | 1.74 (0.33, 13.06) | 0.534 |  | 0.993 |
| 24-32yrs | 0.92 (0.11, 8.06) | 0.936 | 2.69 (0.58, 19.26) | 0.245 |  | 0.992 |
| 33-50yrs | 2.38 (0.49, 17.41) | 0.317 | 2.15 (0.38, 16.51) | 0.399 |  | 0.992 |
| Intensity (epg) | 0.087 (0.65, 1.17) | 0.318 | *1.46 (1.06, 2.14)* | *0.034* |  | 0.367 |
| **SmLy6B** | | | | | | |
|  | **IgG1** | | **IgG4** | | **IgE** | |
|  | **OR (95% CI)** | **p-value** | **OR (95% CI)** | **p-value** | **OR (95% CI)** | **p-value** |
| 10-13yrs | 0.51 (0.16, 1.49) | 0.25 | 1.14 (0.36, 3.71) | 0.830 |  | 0.993 |
| 14-23yrs | 0.85 (0.31, 2.35) | 0.755 | 0.61 (0.18, 2.06) | 0.418 |  | 0.606 |
| 24-32yrs | 0.90 (0.34, 2.43) | 0.836 | 0.89 (0.28, 2.88) | 0.838 |  | 0.390 |
| 33-50yrs | 0.53 (0.18, 1.50) | 0.233 | 1.26 (0.38, 4.29) | 0.700 |  | 0.876 |
| Intensity (epg) | 1.01 (0.84, 1.24) | 0.892 | *1.78 (1.36, 2.44)* | *<0.001* |  | 0.833 |

*Reference group 7-9yrs of age.

Age was divided into the following groups to account for the non-linear relationship between infection intensity and age: 7-9yrs (n=39), 10-13yrs (n= 41), 14-23yrs (n= 42), 24-32yrs (n=44), 38-50yrs (n=45). Pre-treatment antibody responses were classed as a binomial variable: "responders" and "non-responders". Responders were individuals with antibody response greater than the mean + 3 standard deviations of the response of a plasma panel donated by European/North American individuals. Antibody responses were analysed by logistic regression, controlling for age-group and infection intensity. IgE models could not be computed due to some age groups having n value of zero.
