# Supplementary figures and images for: Human IgG1 Responses to Surface Localised Schistosoma mansoni Ly6 Family Members Drop following Praziquantel Treatment
Source: PLoS Negl Trop Dis. 2015 Jul 6;9(7):e0003920. doi: 10.1371/journal.pntd.0003920 (PMC4492491; doi:10.1371/journal.pntd.0003920)

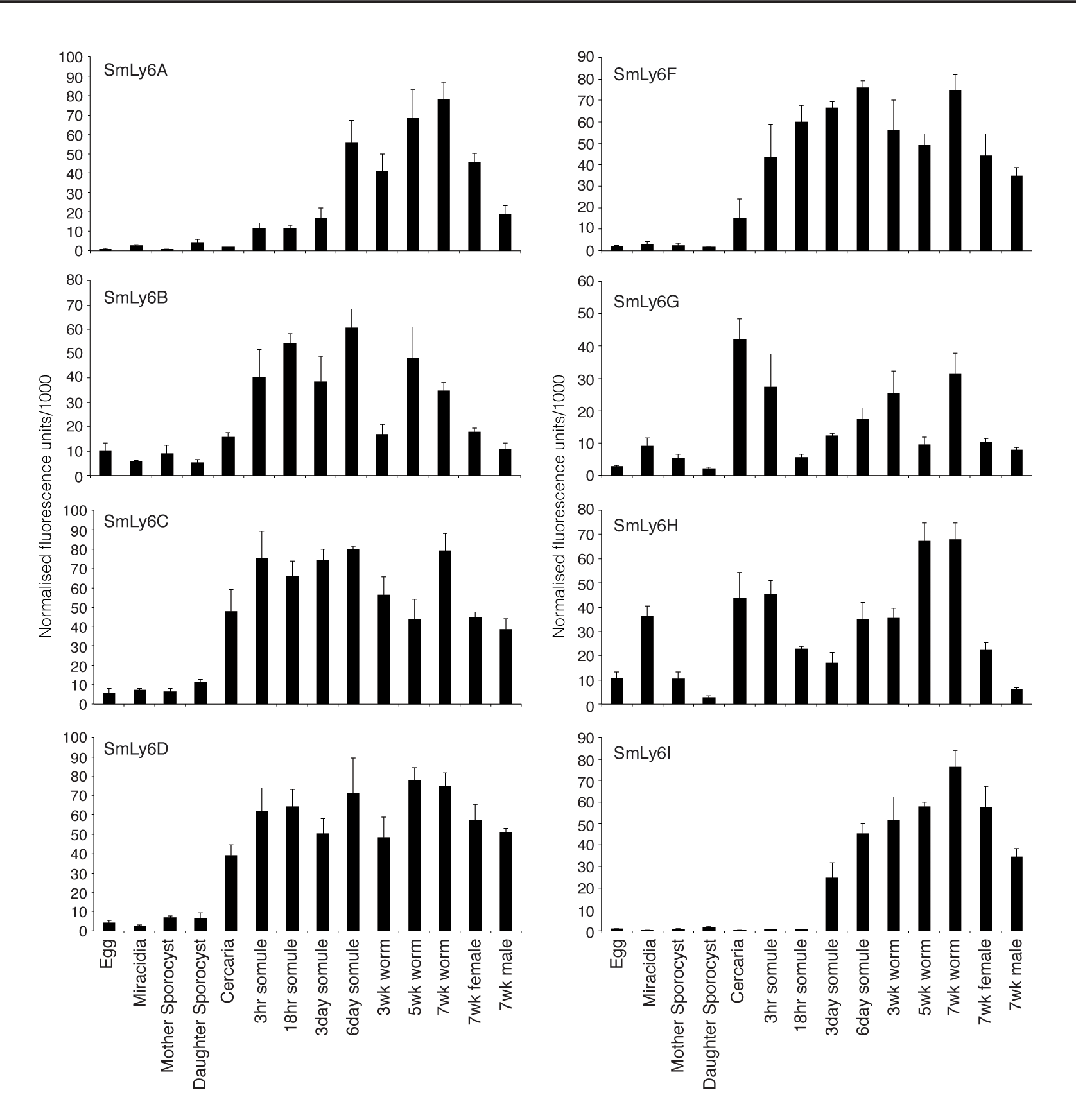

Supplement: S1 Fig — Profiles from the S. mansoni lifecycle DNA microarray data available via Array express [10] under the experimental accession number E-MEXP-2094. Values are mean normalized fluorescence units ± sem. (TIF) [file pntd.0003920.s003.tif]

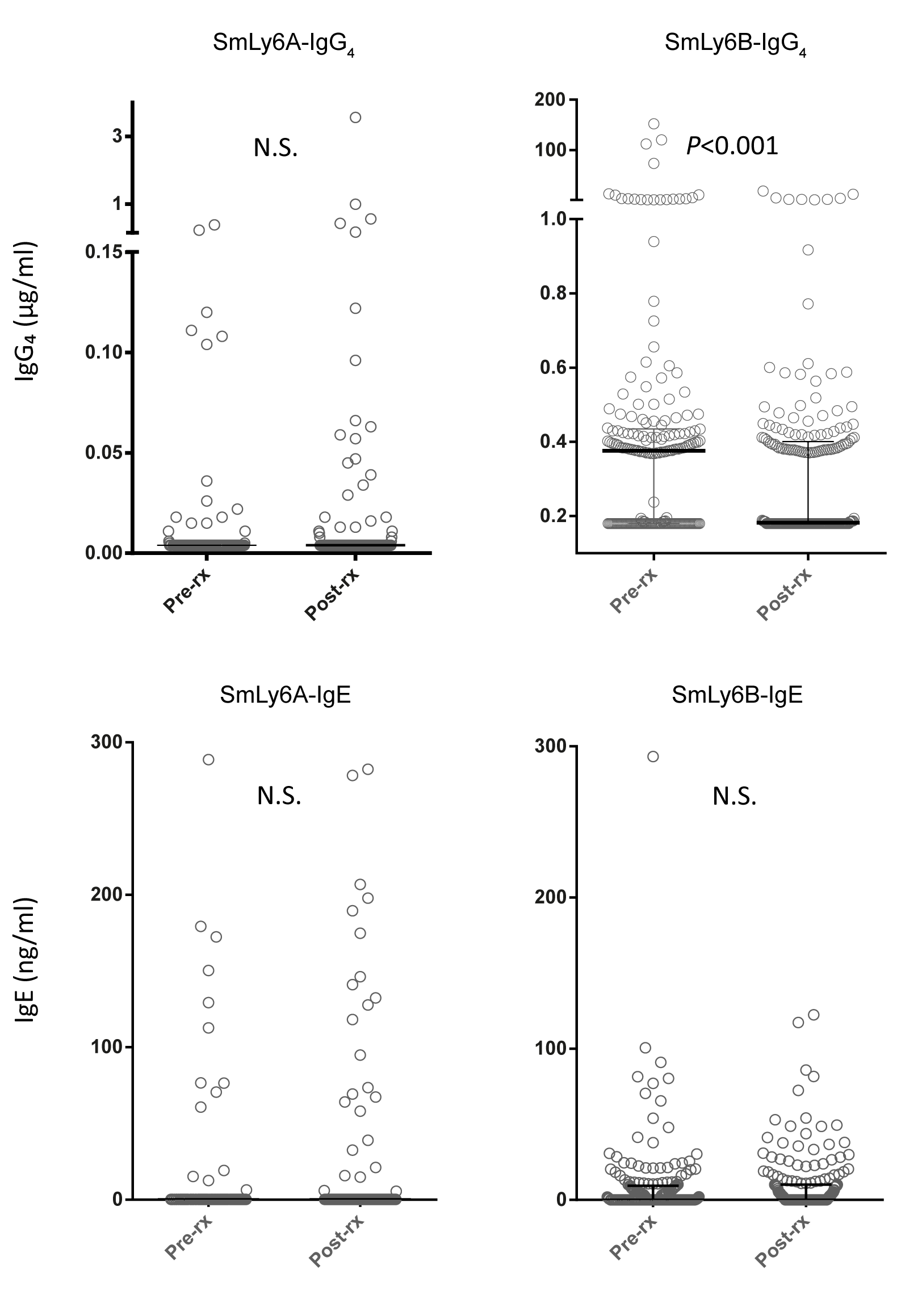

Supplement: S2 Fig — SmLy6A- and SmLy6B-specific IgG4 and IgE were measured before and 9 weeks after praziquantel treatment in a cohort of infected males. Pre and post-praziquantel treatment IgG4 and IgE antibody responses to SmLy6A and SmLy6B, including median value and interquartile range. Statistical analysis was performed using the Wilcoxon Signed Rank test (significance level P<0.05, n = 193). (TIF) [file pntd.0003920.s004.tif]
